# TP53 mutations in ovarian carcinomas from sporadic cases and carriers of two distinct *BRCA1 *founder mutations; relation to age at diagnosis and survival

**DOI:** 10.1186/1471-2407-5-134

**Published:** 2005-10-17

**Authors:** Pedro Kringen, Yun Wang, Vanessa Dumeaux, Jahn M Nesland, Gunnar Kristensen, Anne-Lise Borresen-Dale, Anne Dorum

**Affiliations:** 1 Department of Genetics, The Norwegian Radium Hospital, Montebello, 0310 Oslo, Norway; 2 Department of Gynecologic Oncology, The Norwegian Radium Hospital, Montebello, 0310 Oslo, Norway; 3 University of Oslo, The Norwegian Radium Hospital, Montebello, 0310 Oslo, Norway; 4 Department of Pathology, The Norwegian Radium Hospital, Montebello, 0310 Oslo, Norway; 5 Institute of Community Medicine, University of Tromsø

## Abstract

**Background:**

Ovarian carcinomas from 30 *BRCA1 *germ-line carriers of two distinct high penetrant founder mutations, 20 carrying the 1675delA and **10 **the 1135insA, and 100 sporadic cases were characterized for somatic mutations in the *TP53 *gene. We analyzed differences in relation to *BRCA1 *germline status, *TP53 *status, survival and age at diagnosis, as previous studies have not been conclusive.

**Methods:**

DNA was extracted from paraffin embedded formalin fixed tissues for the familial cases, and from fresh frozen specimen from the sporadic cases. All cases were treated at our hospital according to protocol. Mutation analyses of exon 2 – 11 were performed using TTGE, followed by sequencing.

**Results:**

Survival rates for *BRCA1*-familial cases with *TP53 *mutations were not significantly lower than for familial cases without *TP53 *mutations (p = 0.25, RR = 1.64, 95% CI [0.71–3.78]). Median age at diagnosis for sporadic (59 years) and familial (49 years) cases differed significantly (p < 0.001) with or without *TP53 *mutations. Age at diagnosis between the two types of familial carriers were not significantly different, with median age of 47 for 1675delA and 52.5 for 1135insA carriers (p = 0.245). For cases ≥50 years at diagnosis, a trend toward longer survival for sporadic over familial cases was observed (p = 0.08). The opposite trend was observed for cases <50 years at diagnosis.

**Conclusion:**

There do not seem to be a protective advantage for familial *BRCA1 *carriers without *TP53 *mutations over familial cases with *TP53 *mutations. However, there seem to be a trend towards initial advantage in survival for familial cases compared to sporadic cases diagnosed before the age of 50 both with and without *TP53 *mutations. However, this trend diminishes over time and for cases diagnosed ≥50 years the sporadic cases show a trend towards an advantage in survival over familial cases. Although this data set is small, if confirmed, this may be a link in the evidence that the differences in ovarian cancer survival reported, are not due to the type of *BRCA1 *mutation, but may be secondary to genetic factors shared. This may have clinical implications for follow-up such as prophylactic surgery within carriers of the two most frequent Norwegian *BRCA1 *founder mutations.

## Background

Ovarian cancer is one of the leading causes of cancer-related death in women today. It is the 4^th ^most common cancer in women in Norway and accounts for 5 – 6% of all cancers [1,2]. Mean age at diagnosis for sporadic cases have been reported to 62.3 years [3], and in Norway to 65 years. Age-standardized incidence rates were 13.5 pr 100.000, and close to 40% of the patients is achieving 5-year survival according to The Norwegian Cancer Registry (OVANOR 1991 – 1996).

Almost 10% of epithelial ovarian cancer cases are associated with dominant genetic predisposition, in most cases (80 – 90%), linked to mutations in *BRCA1 *or *BRCA2 *[4-6]. Mean age at diagnosis for these inherited cases have been reported to be from 49 to 54.3 years [3,7]. The penetrance of the disease in mutation carriers varies, and has been reported to be from 27 – 80% [8-10]. It should be noted that both the incidence rate for hereditary cases and the penetrance of the disease may differ depending on geographic and ethnic origin [11]. The survival rate may also vary depending on type and localization of the mutation. Some studies have reported that ovarian cancer patients carrying germ-line *BRCA1 *mutations have an enhanced survival rate compared to sporadic cases [3,12-14]. Other studies demonstrated only an initial survival advantage that disappeared with time, and concluded that no enhanced survival rates follows *BRCA1 *dysfunction [15-17]. These studies predict a survival rate for *BRCA1 *familial ovarian cancer that is equal to or higher than non-familial cases.

Both the penetrance estimates and the survival rates are based on studies in populations with strong founder effects, and may therefore be biased. The type of mutation in the *BRCA1 *gene may affect the timing of the diagnosis of the disease, the response to environmental exposure causing DNA damage, the efficiency of DNA repair, and the frequency of somatic mutations developing in the tumor. These factors may in turn affect the survival rate.

Mutations in the *TP53 *tumor suppressor gene are the most common genetic alteration in human tumors and have been suggested as a molecular marker for prognosis. *TP53 *encodes a nuclear phosphoprotein located at chromosome region 17p13 involved in cell cycle arrest and DNA repair and somatic *TP53 *mutations are known to associate with familial ovarian cancer. In ovarian tumors from *BRCA1 *mutation carriers, somatic *TP53 *mutations are found in 60 – 80% of the cases [18-22]. Thirty to 50% of all ovarian cancers have been reported to harbor a *TP53 *mutation [18-20,23,24]. Further, in 30 – 85% of the sporadic ovarian carcinomas both a *TP53 *mutations and a somatic *BRCA1/BRCA2 *mutation have been found [18,20,25].

These findings implicate that *TP53 *and *BRCA1 *directly interacts and may play an important role in DNA repair processes and tumor suppression [26,27]. However, despite the high frequency of mutations in the tumor suppressor gene *TP53*, there are several reports concluding that *TP53 *is not a good predictor of prognosis in sporadic ovarian cancer patients [24,28,29].

We have previously reported two Norwegian *BRCA1 *founder mutations; 1135insA [30] and 1675delA [11]. Carriers of these mutations show almost the same penetrance for ovarian- and breast cancer and the penetrance is also high compared to most reported *BRCA1 *mutation carriers. By age 50, 48% of mutation carriers had experienced breast- and/or ovarian cancer. Mean age of ovarian cancer diagnosis was ~55 years [10]. Three per cent of all Norwegian ovarian cancers are caused by either of the two founder mutations [31]. As a result of a clinical follow-up program for early diagnosis in women from breast-ovarian cancer kindreds, these two mutations may account for more than half of those with a *BRCA1 *mutation in Norway. The histopathological characteristics of both breast and ovarian cancer indicated an unfavorable prognosis in these mutation carriers [32].

In the present study, we have screened epithelial ovarian tumors from 30 familial cases and 100 sporadic cases for somatic mutations in the *TP53 *gene. The cancer treatment was according to our hospital protocol. The familial cases consisted of one group with the *BRCA1 *1135insA mutation and the other had the *BRCA1 *1675delA mutation [10]. The *TP53 *mutation status was correlated to survival, age at diagnosis and histopathological features.

## Materials

Formalin fixed and paraffin embedded ovarian cancer tissue from 30 *BRCA1 *germ line mutation carriers were collected and used for DNA extraction. Of the familial cases 20 patients carried the 1675delA mutation and another 10 patients the 1135insA mutation, which is a representative distribution between the two mutations in the Norwegian population. The *BRCA1 *carriers were from families with at least two first-degree relatives, or second-degree relatives through male, with ovarian cancer and/or breast cancer under age 60. All cases were sampled from pedigree regardless of survival status, as ovarian cancer treatment is centralized to our hospital. Analysis of fresh frozen specimen of tumor DNA from the 100 sporadic cases sampled from 1992–2003, included in this study has previously been reported [29]. Both groups were diagnosed and treated at the Norwegian Radium Hospital according to protocol. The patient characteristics are shown in Table 1. All tumors were reviewed at our department of pathology, the familial tumors by our team pathologist, and were classified and graded according to the World Health Organization (WHO) criteria. Follow-up time for each case was calculated from the date of diagnosis up to date of death or end of study (15^th ^April, 2004).

## Methods

### DNA extraction and *TP53 *mutation analysis

DNA was manually extracted from paraffin-embedded tissue sections of tumor material using 5 sections of 10 μ. A modification of the procedure described by Miller [33] was used. The modification included using as much as possible of the top water layer of the 700 ml DNA/lysis buffer and 1 ml phenol/chloroform/water mix, and repeating the extraction step once. The protocol was optimized to give high yield of good quality DNA.

Mutation analyses of exons 2–11 of the *TP53 *gene in the 30 cases with *BRCA1 *germ line mutations were performed by TTGE followed by sequencing. Primers, PCR conditions and gel running conditions were as described elsewhere [34]. Samples with aberrantly migrating bands on TTGE were isolated, submitted to a new PCR and sequenced. Analysis of the fresh frozen specimen of tumor DNA from the 100 sporadic cases has previously been reported [29].

### Statistical analyses

In univariate analyses, a log rank test have been used to investigate the effect of age at diagnosis, *BRCA1 *and *TP53 *mutations on the survival rate. In multivariate analyses, Cox proportional hazards regression analysis was used. Hazard ratios (HR's) are given with 95% confidence intervals (CI's). Statistical significance rates were set at 0.05. The software SAS^® ^version 8.2 was used for statistical analyses.

## Results

### *TP53 *characterizations and novel mutations

Nineteen of the 30 ovarian carcinomas showed one or more aberrant migrating bands on TTGE in one or more exons and was sequenced (Table 2). A total of 21 sequence changes were detected. Two cases had two different *TP53 *sequence changes in their tumors, one being a silent mutation. Nine mutations were missense mutations, four nonsense, three were silent sequence changes (not previously reported as polymorphisms) and two were intronic sequence changes of unknown function.

The frequency of transitions vs. transversion in this hereditary cohort (85.7% and 14.3%) was also quite similar to that reported in the IARC database for sporadic cases (88% and 12%), but differed slightly from the sporadic cases in this study (76.4% and 23.6%). The frequency of mutations likely to cause protein alteration were 68.0% (68/100) for the sporadic cases and 53.3%(16/30) for the familial cases. The *TP53 *mutation frequency in the two different *BRCA1 *carriers differed slightly with 11/20 (55.0%) in the *BRCA1 *1675delA carriers and 5/10 (50.0%) in the *BRCA1 *1135insA carriers. The 1675delA carriers had 7.7% transversions and 92.3% transitions while the 1135insA carriers had 12.5% transversion and 82.5% transitions. Four of the *TP53 *mutations were novel and not previously reported in ovarian cancer in the IARC *TP53 *Database [35] or the SOUSSI database. These mutations affected codon 205 (tyr>ser), 260 (ser>ser), 267 (arg>gln) and 293 (gly>arg). All mutations detected resided in exons 5–8. When comparing the *TP53 *mutation spectrum in these familial cases with that of ovarian cancers cases reported in the IARC database and to the 100 sporadic ovarian cancer cases with a *TP53 *mutation, no obvious differences were seen either with respect to exon distribution or codon wise (data not shown), although a slightly lower frequency of mutations in exon 5 and a slightly higher in exon 8 were seen in the hereditary cases. The *TP53 *mutations in the 100 sporadic cases used in this study is reported elsewhere [29].

### Age at diagnosis, survival, *BRCA1 *and *TP53 *status

Median age at diagnosis among sporadic cases and familial cases that carried 1675delA or 1135insA mutations is presented in Table 1. As expected, the familial cases are diagnosed earlier in life than sporadic cases (p < 0.001). The difference in median age of onset between the 1135insA and 1675delA mutation carriers was not significant (p = 0.245).

In the univariate analysis of the combined group, neither *BRCA1 *status nor age at diagnosis was significantly associated to survival (p = 0.87 and p = 0.50 for *BRCA1 *status and age at diagnosis (categorized into < 50 and ≥ 50 years), respectively). *TP53 *mutation did not significantly reduce the survival rates (p = 0.35). Notably, interaction between *BRCA1 *status and *TP53 *status was borderline significant (test for interaction: p = 0.06) while the one between *BRCA1 *status and age at diagnosis was statistically significant (test for interaction: p = 0.05). We further analyzed these factors adjusted for tumor grade, however, results did not substantially change (test interaction: p = 0.04 and p = 0.05 for BRCA1*TP53 and BRCA1*age at diagnosis, respectively).

No association between age at diagnosis and survival time was found among sporadic cases (p = 0.88). Familial cases with late age at diagnosis (≥50 years) had a slightly higher risk of dying than the cases with an early age at diagnosis, however the association did not reach significance, possibly due to a lack of statistical power (RR = 1.65, 95% CI [0.79–3.43], p = 0.14). Among cases diagnosed at age 50 years or more, familial cases had a trend towards a higher risk of dying than sporadic cases (RR = 1.75, 95% CI [0.93–3.30], p = 0.08). After adjustment for the effect of tumor grade and *TP53 *status (RR = 1.80, 95% CI [0.94–3.43], p = 0.08) (data not shown). Table 3 shows the risk ratios associated to *TP53 *mutations after stratification for *BRCA1 *status.

There was no significant difference in survival observed among *TP53 *mutations carriers compared to non-*TP53 *mutations carriers, neither for the familial nor the sporadic cases (Log-rank test for *TP53 *in familial cases: p = 0.25 and log-rank test for *TP53 *in sporadic cases: p = 0.88) (Table 3).

## Discussion

Some studies have reported an enhanced survival for *BRCA1 *carriers with ovarian cancer compared to sporadic cases [12-14,36,37], but these studies have not taken *TP53 *status in the tumors in to consideration. Other studies in which *TP53 *status have been included concludes that there is no difference in survival [16].

Our results do not show an enhanced survival rate for familial cases compared to sporadic cases, even after adjustment for *TP53 *status when all age groups were included. Further, no significant difference in survival rates was observed between the familial cases with and without *TP53 *mutations (Table 3).

These results **do not **support earlier observations regarding the importance of the p53/BRCA1 interaction on cell proliferation and ovarian carcinogenesis. Most penetrance estimates and survival rates are based on studies in populations with strong founder effects, and may therefore be biased [15-17,38]. Two Ashkenazi founder mutations occur in *BRCA1 *185delAG and 5382insC (carrier frequencies of 0.9% and 0.13%), with mean age at diagnosis 54 years. How the type of mutation in the *BRCA1 *gene affects survival, age at diagnosis of the disease, the response to environmental exposure causing DNA damage, and the efficiency of DNA repair, is not clarified. Heterozygote advantage or an increase in biological fitness conferred on carriers of a disease causing mutation (like *BRCA1*?), often a resistance to certain infections that were common in times past, can cause an increase in allele frequency [39]. Genetic factors with impact on survival and age at onset of disease, to after childbearing age, would be preferential.

The trend for an increased survival in favour of the early age at onset in familial cases compared to late age at onset in familial cases may be attributed to younger patients having greater physical strength, less somatic mutations, and manage illness better than older patients. On the other hand, one might also expect this trend in sporadic cases, which was not the case. One limitation of our study is the small numbers of *BRCA1 *carriers. In our study, the statistical power to detect *BRCA1 *effect was 76%.

Consequently, our findings should be confirmed in larger studies. The conflicting literature on the impact of *BRCA1 *mutation status on ovarian cancer survival should promote additional studies from different ethnic populations, and thereby allow investigators to study whether or not there is a survival benefit due to *BRCA1 *mutation, or may be secondary to other common inherited genetic factors, which may be shared in ethnic or geographic isolated populations.

Alterations in the *TP53 *gene have been shown to affect breast cancer survival and in particular patients with mutations in the zinc-binding domains have poor survival [40]. In sporadic ovarian carcinoma several studies reports that no or little effect of *TP53 *mutations have been seen [17,24,28], which is similar to the results reported here. *TP53 *alterations are also suggested to alter ovarian cancer survival in *BRCA1 *germ line patients [13,14], while other groups concludes with a failure of *BRCA1 *dysfunction to alter ovarian cancer survival [16]. It should also be noted that a considerable fraction (60–80%) of all familial *BRCA1 *ovarian cancers harbor *TP53 *mutations [18,19,21,22]. Only a few studies have reported analysis of *TP53 *mutations in relation to *BRCA1 *associated ovarian cancer [20,41]. The present study is the first investigating somatic *TP53 *mutations in ovarian tumors from carries of two distinct high penetrant *BRCA1 *germ-line mutations, relating it to survival and age at diagnosis of disease and compares it to sporadic cases. We have previously studied the distribution in age at diagnosis in *BRCA1 *carriers and non-carriers as a part of a cohort study. Three percent of Norwegian ovarian cancers are caused by *BRCA1 *1675delA or 1135insA [31,42], with a distribution similar to that found in this study (Table 1). Further, Bjørge et al. [43] found that 87.0% of Norwegian sporadic ovarian cancers was papillary serous adenocarcinoma, an aggressive histo-prognostic factor.

Eigthy percent of both familial and sporadic ovarian cancer cases in this study were papillary serous adenocarcinoma. Questions need to be addressed concerning the clinical effects of mutations in the *BRCA1 *gene, why some mutation carriers develop breast cancer, others develop ovarian cancer, and some develop both. We do not know whether the cancers occurring in mutation carriers are significantly different from those occurring in non carriers. The frequency of *TP53 *mutations in the familial cases altering the protein was 53.3%, which is somewhat higher than other studies of familial *BRCA1 *ovarian cancer (31–50%) [18-22,24,25]. Although the number of familial cases in this study is limited, a slightly higher frequency of mutations was found in exon 8 and a lower frequency in exon 5 compared to sporadic cases in the IARC database. The same tendency has been reported by others [20]. However, a non-significant difference in *TP53 *mutation frequency was observed between the familial and sporadic cases in this study.

Of the novel mutations found in the familial cohort the codon 205 mutation has previously been reported in several other tumors like head and neck SCC as well as breast- and colorectal carcinoma. The amino acid change in codon 255 and 293 are only reported once, in oesophageal SCC and bladder cancer, respectively. The silent codon 260 mutation are reported in two different cancer tissues; lung (SCLC) and colorectal carcinoma. Environmental exposure, both external and internal, is known to influence the spectrum of mutations. Whether hormonal disturbance may affect the mutation rate and spectrum is not known, but if so, it may be expected that *BRCA1 *carriers are more sensitive to such exposure.

## Conclusion

Interestingly, no difference in survival was observed between *TP53 *mutation carriers among the familial carries or among the sporadic cases (Table 3). Further, we did not find an overall difference in survival between familial *BRCA1 *carriers and sporadic epithelial ovarian cancer cases, even after adjustment for *TP53 *status. For cases diagnosed over the age 50 there was a trend toward higher survival for sporadic cases.

## List of abbreviations

TTGE; temporal temperature gradient gel electrophoresis; PCR, polymerase chain reaction; FIGO, International Federation of Gynaecology and Obstetrics. RR; risk ratio.

## Competing interests

The author(s) declare that they have no competing interests.

## Authors' contributions

PK participated in the design of the study, carried out the molecular genetic studies, sequence alignment and drafted the manuscript. YW participated in screening of the sporadic cases and sequence alignment. VD performed the statistical analyses. GK performed clinical updates of sporadic cases.

JMN evaluated pathology sections of sporadic and familial cases. ALBD conceived the study, participated in its design and helped draft the manuscript. AD conceived the study, performed clinical dates of the familial cases and helped draft the manuscript. All authors read and approved the final manuscript.

## Pre-publication history

The pre-publication history for this paper can be accessed here:



## Figures and Tables

**Table 1:** Patient characteristics

	Sporadic cases	*BRCA1 *carriers		
		
		All familial cases	1135insA	1675delA
No of cases	100	30	10	20
Age at diagnosis: Median	59	49	52.5	47
range	39 – 80	39 – 80	41 – 80	39 – 65

FIGO stage				
I – II	9 (9.0%)	8 (26.7%)	2 (20.0%)	6 (30.0%)
III	65 (65.0%)	14 (46.7%)	5 (50.0%)	9 (45.0%)
IV	26 (26.0%)	8 (26.7%)	3 (30.0%)	5 (25.0%)

Histology				
Serous	82 (82.0%)	24 (80.0%)	7 (70.0%)	17 (85.0%)
Mixed	7 (7.0%)	2 (6.6%)	1 (10.0%)	1 (5.0%)
Endometroid	5 (5.0%)	3 (10.0%)	1 (10.0%)	2 (10.0%)
Unclassified	6 (6.0%)	1 (3.3%)	1 (10.0%)	0

Grade of differentiation				
1	7 (7.0%)	0	0	0
2	26 (26.0%)	5 (16.6%)	1 (10.0%)	4 (20.0%)
3	67 (67.0%)	24 (80.0%)	9 (90.0%)	15 (75.0%)
Unknown	0	1 (3.3%)	0	1 (5.0%)

Survival >5 years	23 (23.0%)	10 (33.3%)	3 (30.0%)	7 (35.0%)

*TP53 *mutation status	72.0%	53.3%	50.0%	55.0%

All tumors are epithelial adenocarcinomas

**Table 2:** *TP53 *mutations, survival and histopathological features for each case.

Case	^a^*BRCA1 *carrier type	Stage	^b^Grade	Type	TTGE	Exon	Codon	Mutation	aa change	Age of diagnosis	^c^Survival months	^d^Vital status
3453	1	3	2	ser	pos	5		559+1G>A	splice	53	83	1
**10**	1	3	3	ser	pos	6	213	CGA>TGA	arg>stop	65	22	**3**
2857	1	3	3	ser	pos	6	213	CGA>TGA	arg>stop	53	83	3
**4**	1	4	3	ser	pos	6	216	GTG>ATG	val>met	59	11	**3**
**27**	1	2	3	endo	pos	7	237	ATG>ATA	met>ile	39	61	**3**
**8^e^**	1	3	3	ser	pos	7	260	TCC>TCT	ser>ser	49	84	**3**
						8	306	CGA>TGA	arg>stop			
**14**	1	1	3	ser	pos	8	267	CGG>CAG	arg>gln	51	25	**3**
**13**	1	4	3	ser	pos	8	273	CGT>AGT	arg>ser	39	15	**3**
**29**	1	3	3	mix	pos	8	273	CGT>TGT	arg>cys	50	36	**3**
2842	1 1	4	3	ser	pos	8	273	CGT>TGT	arg>cys	39	35	3
**21**	1	3	3	ser	pos	8	293	GGG>AGG	gly>arg	39	21	**3**
**26**	1	3	2	ser	pos		intron	G>A	ivs5	53	93	**1**
**9**	1	4	2	ser	neg					50	39	**3**
**11**	1	3	3	ser	neg					44	8	**3**
**17**	1	3	3	ser	neg					47	30	**3**
**24**	1	2	4	ser	neg					59	20	**3**
**34**	1	1	3	ser	neg					46	120	**1**
**30**	1	1	2	ser	neg					43	199	**2**
**32**	1	2	3	ser	neg					48	108	**1**
**3351**	1	4	3	endo	neg					44	9	**3**
**1**	2	4	3	ser	pos	5	144	CAG>TAG	gln>stop	41	1	**3**
**3**	2	3	3	uncl	pos	6	196	CGA>TGA	arg>stop	48	46	**3**
**20**	2	3	3	ser	pos	6	205	TAT>TCT	tyr>ser	52	36	**3**
**28**	2	4	3	ser	pos	7	261	AGG>AGA	arg>arg	80	12	**3**
**7^e^**	2	3	3	ser	pos	7	255	ATC>GTC	ile>val	58	30	**3**
						5	141	TGC>TGT	cys>cys			
**12**	2	1	3	ser	pos	8	280	AGA>GGA	arg>gly	49	134	**1**
**15**	2	3	3	ser	pos		intron	C>T	ivs7	50	19	**3**
**5**	2	4	3	ser	neg					60	45	**3**
**18**	2	2	3	endo	neg					44	117	**3**
**22**	2	3	2	mix	neg					47	96	**3**

a: 1 = 1675delA and 2 = 1135insA. b: undifferentiated (4). c: all patients were followed until diseased or to 15^th ^April, 2004. d: alive without cancer (1), alive with cancer (2), and dead by cancer (3). e: two different mutations detected in sample.

**Table 3:** Hazard risk of *TP53 *mutations on familial and sporadic ovarian cancer cases.

	# cases	HR	CI (95%)	p-value
Familial Cases	30			
*TP53 *no mutation	14	1 (ref)		
*TP53 *mutation	16	1.64	0.71 – 3.78	0.25
Sporadic cases	100			
*TP53 *no mutation	26	1 (ref)		
*TP53 *mutation	76	0.96	0.55 – 1.66	0.88
